# The implications and prospect of cuproptosis-related genes and copper transporters in cancer progression

**DOI:** 10.3389/fonc.2023.1117164

**Published:** 2023-02-28

**Authors:** Qianwen Zhao, Tonggang Qi

**Affiliations:** Institute of Medical Sciences, The Second Hospital of Shandong University, Jinan, China

**Keywords:** cancer, cuproptosis, cuproptosis-related genes, copper transporters, non-apoptotic regulatory cell death

## Abstract

Currently, cancer has become one of the major public health problems worldwide. Apoptosis is an important anti-cancer defense mechanism, which is used in the development of targeted drugs. Because cancer cells have endogenous resistance to apoptosis,the clinical efficacy of related drugs is not ideal. Therefore, non-apoptotic regulatory cell death may bring new therapeutic strategies for cancer treatment. Cuproptosis is a novel form of regulatory cell death which is copper-dependent, regulated and distinct from other known cell death regulatory mechanisms. FDX1,LIAS,and DLAT named cuproptosis-related genes play an essential role in regulating cuproptosis. Meanwhile, abnormal accumulation of copper can be observed in various malignant tumors. The correlation has been established between elevated copper levels in serum and tissues and the progression of several cancers. Copper transporters, CTR1 and Copper-transporting ATPases(ATP7A and ATP7B), are mainly involved in regulating the dynamic balance of copper concentration to maintain copper homeostasis. Thus,cuproptosis-related genes and copper transporters will be the focus of cancer research in future. This review elaborated the basic functions of cuproptosis-related genes and copper transporters by retrievalling PubMed. And then we analyzed their potential relationship with cancer aiming to provide theoretical support and reference in cancer progression, diagnosis and treatment for future study.

## 1 Introduction

Cancer has become one of the major public health problems worldwide. According to the 2022 Cancer Progress Report released by the American Association for Cancer Research (AACR), there are 18.1 million new cancers globally in 2020, resulting in 9.89 million patients deaths. It is estimated there will be 28 million new cancers worldwide by 2040 (1). Indeed, the fundamental purpose of cancer therapy is to selectively reduce cancer cells and induce cancer cell death. Therefore, researchers have never stopped exploring and studying any type of cell death.

Cell death is a conservative phenomenon that prokaryotic and eukaryotic cells fail to maintain basic life functions.Generally speaking, cell death is divided into accidental cell death (ACD) and regulated cell death (RCD). ACD is an uncontrollable biological process, while RCD has precise molecular mechanisms and complex signal regulation pathways (2). At present, there are dozens of RCD, in which cell apoptosis, pyroptosis, autophagy, ferroptosis and necroptosis have been extensively studied (3). Apoptosis is a pivotal anti-cancer defense mechanism, which is applied to the development of targeted drugs. Because cancer cells have endogenous resistance to apoptosis, the clinical therapeutic effect of relevant drugs is not ideal (4). Thus, non-apoptotic regulatory cell death may bring novel therapeutic strategies for cancer treatment.

In 2022, Tsvetkov et al. (5) proposed a new form of regulatory cell death called “cuproptosis” in Science. Cuproptosis is triggered by the direct combination of copper ions with the lipoacylation component in the tricarboxylic acid (TCA) cycle.This leads to the aggregation of lipid-acylated- related proteins and downregulation of iron-sulfur cluster proteins,eventually resulting in protein toxicity stress and cell death.The team found ten genes involved in the cuproptosis through the whole genome CRISPR-Cas9 function deletion screening.There are seven positive regulatory genes (ferredoxin 1(*FDX1*), lipoic acid synthase (*LIAS*), lipoyl transferase 1 (*LIPT1*), drolipoamide S- acetyltransferase (*DLAT*), dihydrolipoamide dehydrogenase (*DLD*), pyruvate dehydrogenase E1 subunit alpha 1 (*PDHA1*), pyruvate dehydrogenase E1 subunit beta (*PDHB*))and three negative regulatory genes (metal-regulatory transcription factor-1 (*MTF1*),glutaminase (*GLS*), and cyclin- dependent kinase inhibitor 2A (*CDKN2A*)). Simultaneously, there is abnormal accumulation of copper in multiple malignant cancers (6–8). A significant role has been played in promoting tumor cell proliferation, metastasis and angiogenesis by copper (7, 9). All this suggests unbalanced copper homeostasis has an impact on the tumor. Copper transporters 1(CTR1) and copper-transporting ATPases(*ATP7A* and *ATP7B*) participate in the transport and distribution of copper to maintain copper homeostasis (10, 11). Therefore, it is of great significance to study the implication of cuproptosis-related genes and copper transporters in the development of cancer. In this study, we retrieved the functions of three key cuproptosis-related genes (*FDX1*, *LIAS*, *DLAT*) and copper transporters (CTR1,*ATP7A* and *ATP7B*).Then we analyzed their potential relationship with cancer so as to provide theoretical support and reference for future research on cancer progression,diagnosis and treatment.

## 2 Cuproptosis-related genes and cancer

Actually, Tsvetkov et al. (12) in 2019 found the copper ionophore elesclomol-Cu^2+^ complex entered cells and *FDX1* reduced Cu^2+^ to Cu^+^,which ultimately led to a unique copper dependent cell death and could not be blocked by apoptosis inhibitors or ferroptosis inhibitors.However, the mechanism was unclear. In March 2022, based on the above research, Tsvetkov et al. (5) named this copper- dependent cell death as”cuproptosis”. This study demonstrates cuproptosis is closely related to the TCA cycle. Through genome wide CRISPR-Cas9 function deletion screening, it is found that the necessary condition for copper binding is protein lipoylation. And *FDX1* is the upstream regulator of protein lipoylation. By knocking down the single genes *FDX1* and *LIAS*, the deletion of *FDX1* and *LIAS* could enhance the resistance to copper-induced cell death. Thus, *FDX1* and protein lipoylation are the key regulators of cuproptosis. Additionally, the combination of copper with lipoylated protein *DLAT* will induce unstable expression of iron-sulfur cluster protein causing protein toxicity stress and cell death. This brand-new form of non-apoptotic regulatory cell death will be a hot spot for cancer treatment in the future. As three key genes in cuproptosis, *FDX1*, *LIAS*, and *DLAT*, are expected to be the targets in cancer therapy. Next, I will summarize the functions of these genes and their current research on cancer.

### 2.1 FDX1

Iron–sulfur cluster proteins are a kind of protein cofactors that modulates various biochemical processes, including mitochondrial respiration, electron transfer, redox catalysis and biosynthesis (13, 14). The human genome contains two homologous ferredoxins, ferredoxin 1 (*FDX1*) and ferredoxin 2 (*FDX2*). *FDX1* produces a market effect on steroidogenesis, while *FDX2* is involved in the synthesis of Fe -S cluster and heme A (15).

One of the signs of cancer is cellular metabolism disorders (16). It happens that the main function of *FDX1* is to regulate substance metabolism. Hence, researchers have explored the association between *FDX1* and cancer. Downregulation of *FDX1* significantly changes glucose, amino acid, and fatty acid oxidative metabolism in lung adenocarcinoma. Meanwhile, the prognosis of lung adenocarcinoma patients with low expression of *FDX1* is even worse (17). This is consistent with the previous studies.The most common manifestation of lung cancer metabolism is abnormal glucose and lactic acid metabolism (18). Besides, the steroid synthesis regulated by *FDX1* may be related to the occurrence of tumors. *FDX1* is involved in regulating steroid hormone synthesis, receiving electrons transferred from NADPH *via* Adx reductase, which in turn reduces members of the mitochondrial cytochrome P450 (CYP450). CYP450 mainly includes CYP1, CYP2 and CYP3 families. CYP1B1 in the CYP1 family catalyzes the conversion of estradiol to 4-hydroxy-17-β-estradiol (4-OHE2) (19). The metabolite of estradiol,4-OHE2, is involved in the development of some hormone-related cancers. For example, 4-OHE2 undergoes the redox cycle, which produces reactive oxygen species (ROS) and chemoreactive estrogen semiquinone, thereby promoting the occurrence of breast cancer (20). In endometrial cancer, 4-OHE2 participates in the occurrence of endometrial cancer by inducing PTEN mutation on codon 130/131 (21). So the potential association between *FDX1* regulating CYP450 and the pathogenesis of hormone-related cancer deserves further study. From this, we speculate *FDX1* may be involved in the metabolic disorder during the occurrence and development of cancer, subsequently changing its phenotype and tumor microenvironment.

With the proposal of cuproptosis, a research conducted systematic bioinformatic database analysis on *FDX1*. Firstly, the expression of *FDX1* at mRNA and protein levels was reduced in most cancers, such as breast invasive carcinoma (BRCA), colon adenocarcinoma (COAD), lung adenocarcinoma (LUAD), thyroid carcinoma (THCA), etc. Secondly, the expression of *FDX1* was related to the clinical features, anti-tumor drug sensitivity and drug resistance in some cancer types.Simultaneously, gene-set enrichment analysis showed tight correlation between *FDX1* and immune-related pathways, immune cell infiltration, and immunoregulatory genes (22). Although the analysis of public bioinformatic database has some limitations due to the limited clinical and pathological information, it also indicates *FDX1* is expected to become a potential target for cancer treatment and a biomarker for evaluating prognosis in the future.

### 2.2 LIAS

Lipoic acid is an antioxidant synthesized in mitochondria. It can not only clear free radicals,but also participate in regulating mitochondrial energy metabolism and oxidative stress. Lipoic acid synthase (*LIAS*) belongs to the biotin and lipoic acid synthase family, which is involved in catalyzing the final step of lipoic acid synthesis (23). Relevant studies have shown the lower expression of *LIAS* can promote the progression of diseases such as diabetes, atherosclerosis and neonatal epilepsy by increasing oxidative stress (24–26). Downregulating *LIAS* by RNA interference may lead to redox imbalance, mitochondrial dysfunction and inflammation (27). As a result, *LIAS* is related to mitochondrial energy metabolism and antioxidant defense. Mitochondria participate in electron chain transfer and oxidative phosphorylation through TCA cycle. Some tumors with high level of oxidative phosphorylation (such as breast cancer, melanoma, cholangiocarcinoma, etc) are mainly powered by it.Studies have applied drugs targeting oxidative phosphorylation such as PGC-1α to inhibit the proliferation of tumor cells (28–30). Therefrom, we surmise *LIAS* may become a latent therapeutic target for mitochondrial respiration-dependent tumors. Moreover, experiments have proved the mutation of *LIAS* inhibits the activity of proline hydroxylase (PHDs), thereby promoting the activation of *HIF-1* (31). It is universally acknowledged that the higher expression of *HIF-1* is a prerequisite for tumor cell proliferation and migration, angiogenesis and epithelial-mesenchymal transition (EMT) (32). Consequently, *LIAS* may have an effect on tumor proliferation and invasion by regulating *HIF-1*. Finally, the effect of *LIAS* on oxidative stress and inflammation provides clues to its role in tumor immunotherapy. In the atherosclerotic mouse model with the overexpression of *LIAS*, it was found that the number of Treg was increased and T cell infiltration was reduced (25). Similarly,in pulmonary fibrosis mice with the overexpression of *LIAS*, the inhibition of NF-kB relieved the chronic inflammatory response, showing an increase in the number of Treg cells and a decrease in T cell infiltration (33). The latest pan-cancer analysis of the bioinformatics database on *LIAS* is consistent with the above experimental conclusions. Researchers found the expression of *LIAS* was correlated with the infiltration of immune cells (34). In the tumor microenvironment, increased Treg cells will inhibit the activation and function of effector T cells, resulting in the immune escape of tumor cells (35). In conclusion, *LIAS* may have far-reaching effect on the proliferation, angiogenesis and immune escape of tumor.

### 2.3 DLAT

Pyruvate dehydrogenase complex (PDC) is a multienzyme complex located in mitochondria that catalyzes pyruvate, transfers acetyl groups to coenzyme A through *DLAT*, finally transforms to acetyl-CoA. *DLAT* is the E2 subunit of PDC complex, which is critical to TCA cycle (36). So far, a series of studies have demonstrated the role of *DLAT* in tumors. Wen et al. (37)observed the expression of *DLAT* was appreciably upregulated in gastric cancer cells, which promoted oxidative phosphorylation and provide energy for tumor cells by catalyzing the conversion of pyruvate to acetyl-CoA. Shan et al. (38) found *DLAT* promoted tumor cell proliferation and growth by elevating the level of 6PGD lysine acetylation in primary leukemia. According to the research on non-small cell lung cancer, PM2.5 upregulated the expression of *DLAT* to promote glycolysis through the dual regulation mechanism of Sp1-*DLAT* and eIF4E-*DLAT* axis, enhancing the proliferation of tumor cells (39).

Next, we have noticed the association between E4F1 and tumors, which is involved in the modulation of PDC subunits.E4F1 was initially identified as a cellular target of the viral oncoprotein E1A. It regulates *DLAT* transcription to control the metabolism of pyruvate oxidation pathway (40). Most importantly, E4F1 mediates the ubiquitination of tumor suppressor gene *p53*, enhancing its transcriptional activity and eventually arresting the cell cycle (41). Accordingly, E4F1 may affect the development of cancer by coordinating *p53* and *DLAT*.

Finally, *DLAT* is a biological substrate of Sirtuin 4 (SIRT4) lipoamidase activity. SIRT4 induces *DLAT* hydrolysis to regulate TCA cycle by lipoyl- and biotinyl-lysine modifications of *DLAT (* 42). According to the classical theory “Warburg effect”, most tumor cells obtain energy through aerobic glycolysis. It is considered to be a marker of cancer progression and metastasis (43). It was found that the glycolysis was decreased in overexpressing SIRT4 hepatocellular carcinoma (HCC) cell lines. In other words, the overexpression of SIRT4 will reduce the energy obtained by the tumor (44). To sum up, *DLAT* may influence tumor development by regulating pyruvate oxidation, TCA cycle and glycolysis.

## 3 Copper transporters and cancer

On the one hand,Copper is an essential cofactor for enzymes that mediates cell functions. On the other hand, the abnormal accumulation of copper also induce oxidative stress and cytotoxicity (45). Therefore, copper homeostasis is the guarantee to maintain the basic cell functions. Copper transport *in vivo* involves a variety of copper chaperones and copper transporters. Metal reductase STEAP reduces extracellular Cu^2+^to Cu^+^.Then copper transport protein 1 (CTR1) transfers Cu^+^ from outside the cell into it. Copper ions entering the cell are transported through copper chaperone. The leading pathways are as follows: ① Anti oxidant 1(ATOX1) transports copper to trans-Golgi network (TGN) and then combines with copper-transporting ATPases to expel excessive copper ion. ② Copper enters mitochondria through *SLC25A3* for storage.In mitochondria,cytochrome c oxidase 17(COX17) directly delivers copper to cytochrome c oxidase 11 (COX11) and synthesis of cytochrome oxidase protein.COX11 and synthesis of cytochrome oxidase 1(SCO1) are the metallochaperone that inserts copper into the cytochrome c oxidase 1(COX1) and cytochrome c oxidase 2(COX2) respectively. Cytochrome c oxidase assembly factor 6 (COA6) and SCO2 help to maintain the redox balance of SCO1 and in turn its copper binding and delivery to cytochrome c oxidase (COX).Together,these proteins maintain appropriate intracellular copper bioavailability and ensure metallation of copper-dependent enzymes. ③ Copper chaperone for super-oxide dismutase 1(CCS) transports copper to superoxide dismutase 1 (SOD1) so as to remove free radicals (46–50).

Actually,Copper chaperone also has potential role in various tumors. In melanoma, ATOX 1 deficiency reduces MEK levels in copper-binding MAPK signaling and suppresses MAPK signaling activation (51). Li Y et al. (52) found CCS promoted the growth and migration of breast cancer cells by regulating ROS-mediated ERK1/2 activity.In leukemia, COX17 inhibition can increase the level of mitochondrial copper, thereby affecting methionine metabolism and DNA methylation, and reduce the activity of leukemia stem cells (53). SCO2 may synergisticallyinfluence tumor progression by promoting mitochondrial respiration (54).

Next, I will systematically summarize the role of CTR1 and Copper-transporting ATPases in tumorigenesis and development.

### 3.1 CTR1

CTR1 is a high-affinity copper transporter encoded by *SLC31A1*. The C terminus of CTR1 is intracellular and the N terminus is extracellular. It is arranged in the form of homotrimer on the cell membrane to facilitate the inflow of copper. In the pregnant mouse model with the deficiency of CTR1, obvious growth and development defects was discovered in embryo. Even worse, some embryos would die *in utero* in the secong trimester of pregnancy. This demonstrates CTR1 is crucial in embryonic development (55).

Actually, studies have shown CTR1 is highly expressed in melanoma, liver cancer, prostate cancer and other human cancer cell lines (56). Subsequently,many reports investigate the effect of CTR1 on tumor progression from tumor-related signaling pathways, angiogenesis and platinum drug resistance. First and foremost, CTR1 mediates copper causing cell signal cascade disorder indirectly, which leads to the occurrence and development of tumors. Abnormal activation of mitogen-activated protein kinases (MAPK) signaling is a classic hallmark of several malignant tumors. The mutation of RAS and BRAF is the most common (57). In 2014, Counter Research Group (9)confirmed downregulating the expression of CTR1 may influence the combination of MEK1 and copper, thereby inhibiting ERK signaling pathway mediated by BRAF and ultimatingly suppressing the tumor growth. In other words, copper modulated by CTR1 regulates BRAF/MEK/ERK signaling pathway indirectly.

Secondly, angiogenesis is the initial process of tumor proliferation and metastasis (58). Earlier studies have indicated copper is a vital element in promoting angiogenesis. It can not only activate the main factors of angiogenesis by *HIF-1*, but also combine with angiogenin to promote angiogenesis (59). In human umbilical vein endothelial cells (HUVECs), the downregulation of CTR1 prevented copper from entering vascular endothelial cells and reduced angiogenesis (60). Another experiment is in line with the above conclusion. Three peptides rich in histidine and methionine are designed in the copper-binding region of CTR1, preventing copper from entering cells and inhibiting angiogenesis (61). Hence,the inhibition of CTR1 causing unbalanced copper may become a new idea for anti-angiogenesis.

Last but not least, studies have deeply explored the relationship between chemoresistance to cisplatin and the expression of CTR1. Platinum drugs are one of the most common chemotherapeutic agents for many solid tumors. Resistance of tumor cells to cisplatin is the key reason for the poor effect of chemotherapy. Studies have revealed CTR1 is the major transporter of platinum drugs (62). In addition, patients who had received platinum chemotherapy with stage III non-small cell lung cancer were selected to study their tumor tissues by immunohistochemistry experiments. The results indicated the high expression of CTR1 had a better response to cisplatin treatment and favourable prognosis (63). In human ovarian cancer cells, cisplatin downregulated the levels of CTR1 in time- dependent and concentration-dependent manner (64). Apparently, CTR1 has the potential to be a new target to overcome platinum resistance.

### 3.2 Copper-transporting ATPases (*ATP7A* and *ATP7B*)

*ATP7A* and *ATP7B* are two homologous isoforms of copper-transporting P-type ATPases. In order to expel excessive copper from the body, *ATP7A* transports copper to the basement membrane of extracellular matrix, while ATP7B transports it out of hepatic cells and into bile. The mutation of *ATP7A* and *ATP7B* will damage the nervous system, leading to Menkes disease and Wilson disease respectively (65). Additionally, *ATP7A* and *ATP7B* also produce a marked effect in tumor progression. First, The members of LOX family modulates extracellular matrix (ECM) remodeling leading to tumor cell migration (66). According to a study applying CRISPR/Cas9 to silence *ATP7A*,the activity of LOX was inhibited in breast cancer and lung cancer cell lines,suppressing these tumor cell growth and migration (67). Meanwhile, KRAS-mutant colorectal cancer obtained high levels of copper through pinocytosis accompanied by the upregulation of *ATP7A (* 68). Furthermore, The combination of *ATP7A* and VEGFR2 can prevent the degradation of VEGFR2 to promote angiogenesis through P62-SQSTM1 pathway (69). It is noteworthy *ATP7A* or *ATP7B* can bind to platinum drugs to expel it from cancer cells, producing chemotherapy drug resistance. In breast cancer cell MDA-MB-231 with *ATP7A* knocked out, the ability of cisplatin to reduce cell proliferation was enhanced. This study indicated the overexpression of miR-148a-3p negatively regulates *ATP7A*, which will increase the sensitivity of breast cancer cells to cisplatin (70). Similarly, targeting *ATP7B* negatively regulated by miR139 will reduce the chemical resistance of ovarian cancer cells to platinum drugs (71). In conclusion, targeting Copper-transporting ATPases,*ATP7A or ATP7B*,has broad application space in overcoming the resistance of tumor cells to platinum drugs.

## 4 Conclusion

**Figure 1:**
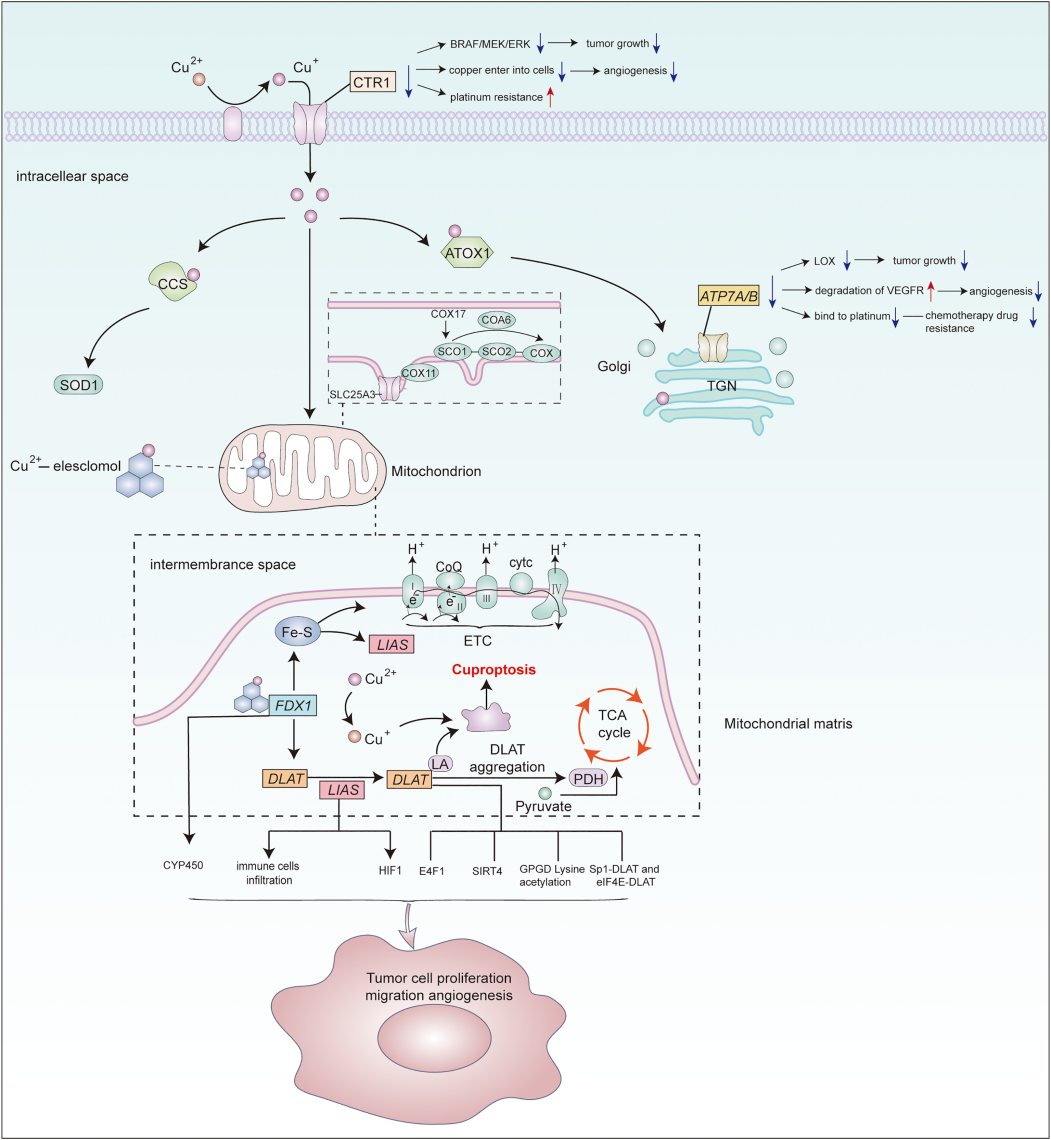
Potential association of cuproptosis–related genes and copper transporters with cancer. Metal reductase STEAP reduces extracellular Cu^2+^to Cu^+^,then CTR1 transfers Cu^+^ from outside the cell into it.Cytoplasmic and mitochondrial metallochaperones (ATOX1, CCS, SCO1, SCO2, COX11, COX17) working in concert to ensure targeted insertion of copper into metalloprotein.In mitochondria,copper enters it through *SLC25A3* for storage.COA6 and SCO2 help to maintain the redox balance of SCO1 and in turn its copper binding and delivery to COX.ATP7A and ATP7B perform copper export function.Copper can induce cuproptosis by binding to lipid-acylated TCA cycle components, promoting lipid-acylated protein aggregation, and inducing protein stress.Vital regulatory genes in cuproptosis or copper homeostasis may regulate various molecules or signaling pathway to affect cancer progression. However, the specifific mechanisms require further study.

Given that copper accumulation occurs in various cancer, it is reasonable to speculate the potential association of cuproptosis–related genes and copper transporters with cancer is multifaceted(**Figure 1**). Based on the finding copper chelator inhibit copper-induced cell death, it is pivotal to deprive appropriate copper to reduce the level of Cu^2+^ under overloaded copper conditions. Specifically,using copper chelator or genetically modifying cuproptosis-related genes and copper transporters might be feasible strategies. Additionally, considering the differences in the abundance and respiratory patterns of lipoylated proteins in tumors, it is necessary to study the precise concentration range leading to cytotoxicity in various disease models,and then establish a reasonable personalized treatment.As cuproptosis was identifified recently, there is no reliable biomarker which limits our ability to determine whether cuproptosis is involved in human pathological. Thus, reliable and sensitive biomarkers of cuproptosis are needed to be identifified in different disease settings. *FDX1*, *LIAS* and *DLAT* are upstream regulatory genes in cuproptosis. At the same time, copper transporters,CTR1,*ATP7A* and *ATP7B*, play a crucial role in copper homeostasis. This review systematically sorted out the functions of the above five key genes.Based on their effects on cancer,we analyzed their intrinsic values in cancer. We have a preliminary summary in **Table 1**. By modulating different substance metabolism,*FDX1*,*LIAS* and *DLAT*, exert impact on tumor cell proliferation, migration, angiogenesis, immune arrest.Specifically,*FDX1*regulates steroid synthesis. *Lias* regulates mitochondrial energy metabolism and oxidative stress. *DLAT* regulates pyruvate oxidation, TCA cycle and glycolysis. These principal upstream regulatory genes in cuproptosis are also of great reference for studying downstream regulatory genes in the future. CTR1 and copper-transporting ATPases mainly influence the resistance of tumor cells to platinum drugs. Regretfully, it is unclear whether their mechanism associated with cuproptosis or not. So far, the research on cuproptosis and its related genes in cancer is limited.It is still at the stage of exploring its connection with cancer,which is lack of fundamental experiment study to prove their cause-and-effect. Undeniably,it provides a research direction with enormous potential for cancer treatment.

**Table 1:** The potential function of cuproptosis-related genes and copper transporters in cancer.

Gene	Potential function	Reference
*FDX1*	*FDX1* may be related to glucose and lactic acid metabolic disorder in cancer. *FDX1* may participate in the steroid synthesis to regulate CYP450 affecting the development of some hormone-related cancers.	(17) (19–21)
*LIAS*	*LIAS* may affect high oxidative phosphorylation cancer by regulating mitochondrial energy metabolism. *LIAS* may have an effect on tumor proliferation and invasion by regulating *HIF-1*. The expression of *LIAS* is correlated with the infiltration of immune cells,which may lead tumor immune escape.	(27–30) (31, 32) (25, 33–35)
*DLAT*	*DLAT* may provide energy for tumor by TCA cycle. *DLAT* may promote tumor cell proliferation by elevating the level of 6PGD lysine acetylation. The regulation of Sp1-*DLAT* and eIF4E-*DLAT* may enhance the proliferation of tumor. *DLAT* may affect cancer by pyruvate oxidation pathway regulated by E4F1. *DLAT* may affect cancer regulated by SIRT4.	(37) (38) (39) (40, 41) (42–44)
*CTR1*	*CTR1* may lead to the occurrence of tumors by copper causing cell signal cascade disorder. *CTR1* may inhibit angiogenesis by preventing copper from entering cells. *CTR1* has the potential to be a new target to overcome platinum resistance.	(51, 52) (55, 56) (9, 57, 58)
*ATP7A* and *ATP7B*	*ATP7A* may suppress tumor cell growth and migration by inhibiting the activity of LOX. *ATP7A* may promote angiogenesis by combining with VEGFR2. *ATP7A* or *ATP7B* can bind to platinum drugs to expel it from cancer cells, producing chemotherapy drug resistance.	(60, 61) (63) (64, 65)

## Author contributions

QZ conceived the framework, searched literature, integrated data, made the table and wrote the manuscript. TQ reviewed the paper and provided fundings. All authors contributed to the article and approved the submitted version.
